# Diagnosis and Management of Parkinson Disease in Individuals with Pre-Existing Mood Disorders

**DOI:** 10.3390/ijerph23020269

**Published:** 2026-02-21

**Authors:** Laura Buyan Dent

**Affiliations:** School of Medicine and Public Health, University of Wisconsin, Madison, WI 53705-2281, USA; dent@neurology.wisc.edu

**Keywords:** Parkinson’s disease, mood disorders, diagnosis, treatment, depression, anxiety, bipolar disorder, neuropsychiatry, multidisciplinary care

## Abstract

**Highlights:**

**Public health relevance—How does this work relate to a public health issue?**

- Parkinson disease and mood disorders are both highly prevalent and increasing globally, and their frequent co-occurrence contributes to delayed diagnosis, treatment complexity, disability, and healthcare utilization.
- Symptom overlap and treatment interactions create systematic risks of misdiagnosis and fragmented care, particularly in settings with limited access to neurology or mental health specialists.

**Public health significance—Why is this work of significance to public health?**

- Earlier recognition of Parkinson disease in people with pre-existing mood disorders can reduce morbidity, prevent inappropriate treatment escalation, and improve functional and quality-of-life outcomes.
- Integrating neurologic and psychiatric care models addresses a major gap in current service delivery for chronic brain disorders and supports more efficient, patient-centered care.

**Public health implications—What are the key implications or messages for practitioners, policy makers and/or researchers in public health?**

- Practitioners and health systems should implement cross-disciplinary screening, referral pathways, and collaborative care models to improve detection and management of combined neurodegenerative and mood disorders
- Policy makers and researchers should prioritize workforce training, access expansion, and research on early biomarkers and integrated interventions to reduce long-term disability and healthcare burden.

**Abstract:**

Parkinson disease (PD) and mood disorders represent two substantial global health burdens that increasingly co-occur as both conditions rise in prevalence worldwide. Diagnosing Parkinson disease in patients with pre-existing mood disorders is clinically challenging due to overlapping symptoms, medication effects, and shared neurobiological mechanisms. Apathy, psychomotor slowing, and fatigue may mimic depressive symptoms, leading to delayed recognition of early parkinsonism. Development of an underlying neurodegenerative disorder could account for some treatment-resistant symptoms or treatment failures if not recognized. Therefore, the identification of PD will change the treatment and management plan significantly. Accurate diagnosis of PD requires a detailed neurologic examination focusing on bradykinesia, rigidity, and resting tremor, supported when appropriate by dopamine transporter imaging (DaT scan) or other emerging biomarkers. Understanding the temporal relationship between psychiatric and motor features helps differentiate prodromal PD from primary mood disorders. Management of patients with both mood disorders and PD integrates dopaminergic replacement therapy for motor symptoms with individualized treatment of psychiatric comorbidities. Levodopa remains the cornerstone for motor control, while dopamine agonists, MAO-B inhibitors, and COMT inhibitors can be added as needed. For depression and anxiety, SSRIs and SNRIs are first-line choices; quetiapine or clozapine are preferred when treatment for psychosis is necessary. Intentional, thoughtful polypharmacy is frequently required. Non-pharmacologic interventions—including cognitive behavioral therapy, structured exercise, and patient–caregiver education—enhance mood, function, and quality of life. Multidisciplinary collaboration between neurology, psychiatry, and allied health professionals is essential for optimal outcomes. This review offers guidance to healthcare providers as well as other interested parties involved in patients with mood disorders who may also be developing or have PD, especially to those who may have limited access to neurologic resources.

## 1. Introduction

Mood disorders are very common worldwide, affecting roughly 17.6% of people in any given year and approximately 29.2% of people over their lifetime. The most common are depression and anxiety, which together impact at least 600 million people globally [1]. Additionally, bipolar affective disorder (BPAD) is increasingly recognized and the prevalence can be as high as 4.4% [2]. Neurodegenerative disorders are also on the rise. Parkinson disease (PD) is second, after Alzheimer disease, as the most common neurodegenerative disease, affecting more than 1% of elderly populations worldwide [3]. Consequently, both mood disorders and neurodegenerative diseases cause a substantial global health burden. The purpose of this paper is to guide practitioners caring for those with mood disorders to recognize and manage their patients developing PD.

Parkinson disease (PD) is a progressive neurodegenerative disorder characterized by motor symptoms—bradykinesia, rigidity, tremor, gait dysfunction—and a broad range of non-motor features. Psychiatric symptoms, especially mood disorders, are among the most common non-motor manifestations of PD, with lifetime prevalence of depression and anxiety exceeding 40% in PD populations [4,5]. Conversely, individuals with mood disorders exhibit an increased risk of later developing PD, pointing to shared neurobiological pathways [6,7]. All of these factors make it important for healthcare providers to recognize and manage these disorders, especially when they coexist. This review provides guidance to healthcare practitioners providing care to patients with mood disorders so they are cognizant of the risk of development of PD, how they may recognize it, and strategies to manage patients in which a mood disorder and PD coexist, especially if access to neurologic care is limited.

## 2. Background and Epidemiology of Parkinson Disease

In 1817, Dr. James Parkinson’s famous “Essay on the Shaking Palsy” was published and described many clinical characteristics of a disease that had been recognized since ancient times [8]. Previously referred to as “paralysis agitans”, this disease entity was increasingly recognized and studied and has now become known as Parkinson disease. The hallmark features are a characteristic resting tremor, rigidity, bradykinesia, and a characteristic “shuffling” gait pattern. The onset is often in mid-life; however, symptoms can be seen in young adulthood and late life, and a juvenile form also occurs [9,10].

In the 1960s, the care of people with PD was revolutionized by the development and discovery of levodopa. When patients with PD were given formulations of levodopa, their symptoms of tremors, rigidity, bradykinesia often markedly improved and some could feel back to near-normal [11,12]. Prior to this treatment, patients often died within a decade or so of diagnosis in an akinetic rigid state. With current treatments, people with PD have an improved lifespan; however, mortality remains increased compared to the general population, with estimates of approximately 60% when adjusted for age and sex [3].

PD is found worldwide and incidence and prevalence increase with age. Epidemiologic studies have shown increasing rates as well. In 2016, 6.1 million individuals worldwide had PD (47.5% women and 52.5% men). This number was 2.4 times higher than in 1990 and attributed to a variety of factors including the increased population of older people, longer lifespans, longer disease duration, and environmental factors/exposures. Prevalence rates were highest in high-income countries [3]. Despite limitations of the studies analyzed, it is clear that clinicians will be seeing increased numbers of people with PD and it will remain a major global healthcare burden.

As the treatment of motor symptoms have improved, the non-motor symptoms have received more attention by clinicians. Among the many non-motor symptoms, depression and anxiety are common and greatly affect the individual’s quality of life. Furthermore, it has been discovered that mood disorders may be considered either a risk factor for developing PD or a prodromal symptom. Additionally, many people with BPAD will also develop PD. Depression, anxiety, and BPAD are highly prevalent and often precede the onset of motor symptoms. Meta-analyses indicate that the prevalence of depression in PD is approximately 38–40%, with anxiety disorders affecting up to 40% of people and bipolar spectrum disorders present in a significant minority, especially in early-onset PD cohorts [13,14,15,16]. These psychiatric symptoms can sometimes manifest more than a decade before the classic motor features of PD become apparent, reflecting early dysfunction in dopaminergic, serotonergic, and noradrenergic systems [6,17,18,19,20]

The temporal relationship between psychiatric symptoms and PD is quantitatively illustrated by large cohort studies. For example, Szatmári et al. found that 16% of PD patients had a psychiatric diagnosis—most commonly mood and anxiety disorders—prior to PD diagnosis, compared to 9.7% in patients with ischemic cerebrovascular lesions (*p* < 0.001) [17]. The risk of developing PD is significantly increased in individuals with pre-existing depression (odds ratio [OR] 1.86, 95% CI 1.81–1.92) and anxiety (OR 1.65, 95% CI 1.57–1.74), as shown in a large population-based study [6].

## 3. Diagnostic Considerations and Complexity

Accurate diagnosis of PD in the context of pre-existing psychiatric comorbidity requires a systematic approach integrating clinical history, neurological examination, and possible ancillary testing in certain clinical situations. Despite many advances, the diagnosis of PD remains a clinical diagnosis at this time. Obtaining a thorough history and performing a complete neurological exam continues to give the best information for making the diagnosis. In 2015, The International Parkinson and Movement Disorder Society published clinical diagnostic criteria for PD to promote objective clinical diagnosis [21,22]. The criteria are based on the presence of bradykinesia (slowness of movement) and at least one additional cardinal sign of rigidity (resistance to passive movement) or resting tremor. When diagnosed by a movement disorders neurologist, the accuracy is approximately 65–84% when compared with autopsy tissue correlation. This variation is attributed to differences in criteria and stage of disease at time of clinical diagnosis [22].

There are many exciting developments in neuroimaging, skin biopsies, genetic testing, and other biomarkers that may provide supportive evidence when appropriately used and interpreted [23,24,25,26,27]; however, an experienced movement disorders specialist remains invaluable. This is especially important when people have pre-existing mood disorders in which some symptoms may overlap. The clinical presentation of PD in patients with pre-existing psychiatric disorders is complicated by substantial symptom overlap. Non-motor symptoms such as apathy, anhedonia, fatigue, and sleep disturbances are common to both PD and mood disorders, making it challenging to distinguish between primary psychiatric illness and PD-related neuropsychiatric manifestations [4,5]. This overlap can result in underdiagnosis or misdiagnosis of PD, particularly in patients with established psychiatric histories, and may delay the recognition of PD by several years [6,17,19]. BPAD presents additional diagnostic challenges, as mood fluctuations, psychomotor agitation or retardation, and cognitive changes can mimic or mask PD symptoms [16].

A detailed history should assess the chronology and evolution of symptoms, focusing on whether psychiatric symptoms are longstanding or have changed in character, severity, or association with emerging motor or autonomic features. The presence of prodromal PD features such as hyposmia, REM sleep behavior disorder, constipation, and subtle cognitive changes should be specifically queried, as these are less typical of primary psychiatric disorders and more suggestive of underlying neurodegeneration [6,7]. Symptoms such as psychomotor slowing, sleep disturbance, fatigue, and cognitive impairment may mimic early parkinsonism or depression, leading to diagnostic delay. Differentiating bradykinesia from psychomotor retardation requires careful neurological assessment. One clue that someone has parkinsonian bradykinesia is that a movement that is repeated will have a decrement in amplitude, almost appearing like fatigue. For example, finger taps or heel taps may begin with a large amplitude, but with repetition the taps will become smaller and may even fuse so that the individual will be unable to separate their fingers or lift their foot from the floor. Also, someone with PD will tend to have variability in the speed of their movements. Psychomotor slowing tends to have a uniformly slow speed for all activities and movements. Additionally, observing facial expression is helpful. Someone with a masked face from PD will demonstrate decreased eye blink rate, have loss of normal spontaneous movements and may appear statue-like. In PD, facial expression will vary; for example, if someone with PD sees a loved one or hears a joke, their facial expression may respond normally, whereas someone with depression may have total absence of facial expression, normal eye blink rate, normal mouth movement and automatic swallowing movements [8,21,23].

Since one of the hallmark features of PD is loss of dopaminergic neurons in the substantia nigra with resultant motor signs and symptoms [9,11,12,23], administering dopamine in the form of levodopa may help when there is diagnostic uncertainty and motor symptoms are present. This levodopa challenge test has not been standardized or validated; however, it may be clinically useful. Levodopa is prescribed in the form of carbidopa/levodopa or benserazide/levodopa and gradually titrated up to 200–300 mg of levodopa three times a day. Significant improvement in rigidity, bradykinesia, and tremor is observed in a “positive” test demonstrating levodopa responsiveness. Although this can be clinically useful and therapeutic, it is highly subjective. Furthermore, if the main symptom is resting tremor, a lack of response does not preclude PD as some PD-related tremors do not respond to levodopa.

Other ancillary tools are available and could provide useful information for diagnostically challenging cases. Although conventional MRI scans are often obtained, they are usually normal for age in people with psychiatric conditions and PD. MRI of the brain may be used to exclude other neurodegenerative disorders or structural lesions, but cannot diagnose PD. However, advanced imaging techniques are being studied to facilitate diagnosis of PD [23,24]. Dopamine transporter single-photon emission computed tomography (DaT scan) imaging can detect nigrostriatal cell loss (sensitivity and specificity ≥90%) and is sometimes used to differentiate PD from essential tremor and drug-induced parkinsonism [23,25,26]. However, a normal DaT scan, implying no nigrostriatal cell loss, does not eliminate the possibility of PD, as false negatives do occur [25,26]. Therefore, the clinical use of DaT scans remains controversial. Nevertheless, there is some evidence that DaT scans may be helpful in distinguishing idiopathic PD from medication-induced parkinsonism in patients with bipolar disorder treated with antipsychotics or lithium [28]. Biomarkers such as alpha-synuclein CSF and blood assays and skin biopsies can provide useful information in certain clinical contexts [23,27]. More biomarkers including DNA tests are being studied as it has been increasingly recognized that there is a prodromal phase of PD [23]. Autopsy studies have shown that by the time motor symptoms of PD develop, over 50% of dopamine-producing neurons in the substantia nigra have been lost [23,29,30]. If reliable and accurate early biomarkers are discovered, then there is hope for development of neuroprotective therapies that can slow or halt loss of dopaminergic neurons. Biomarker tests need to be carefully used and interpreted within the clinical context and ongoing clinical follow up.

## 4. Pathophysiological Links

The pathophysiologic links between PD and mood disorders, particularly depression and anxiety, are multifactorial and involve overlapping neurobiological mechanisms. These include neuroinflammatory processes, neuroimmune dysregulation, and alterations in neurotrophic and stress hormone pathways, dysfunction of multiple neurotransmitter systems (dopaminergic, serotonergic, noradrenergic), and disruption of limbic and cortico-striatal circuits [18,31,32,33]. Chronic neuroinflammation, possibly triggered by environmental factors or α-synuclein pathology, can damage mood-regulating brain regions and contribute to the evolution of mood disturbances [18,32,33]. Alterations in the hypothalamic–pituitary–adrenal axis, neurotrophic factors, and the gut–brain axis have been implicated in the pathogenesis of mood disorders in PD [18,31,33]. Genetic predisposition and psychosocial stressors may modulate vulnerability [30,31,32,33]. Consequently, the risks, causes and clinical course of individuals living with these diseases are quite varied.

PD is characterized by degeneration of dopaminergic neurons in the substantia nigra, but mood disorders in PD are also linked to degeneration in the mesocorticolimbic dopaminergic pathway, as well as serotonergic neurons in the raphe nuclei and noradrenergic neurons in the locus coeruleus. These changes disrupt mood regulation and contribute to depression and anxiety. Additionally, the lateral habenula, which connects dopaminergic and serotonergic systems, may also play a role in mood symptoms in PD [32,33,34]. Functional and structural neuroimaging studies show that mood disorders in PD are associated with abnormalities in limbic circuits, including the anterior cingulate cortex, orbitofrontal cortex, amygdala, thalamus, and ventral striatum. These regions are involved in emotional processing and reward, and their dysfunction correlates with depressive symptoms [18,31,35]. The limbic loop of the basal ganglia, which includes connections between the ventral striatum (nucleus accumbens), ventral pallidum, and prefrontal cortex, is particularly critical for mood regulation and becomes dysfunctional early in PD. This may occur even before motor symptoms are detected [36]. The basotemporal limbic and mediotemporal limbic networks also show disrupted functional connectivity that contributes to both motor and mood symptoms [31,37]. Functional magnetic resonance imaging (fMRI) reveals that between-network connections linking sensorimotor and visual networks may predict motor symptoms, while within-network connections in the insula and sensorimotor network predict mood symptoms, with the middle-to-posterior insula playing a particularly important role in depression and anxiety [38]. Furthermore, studies examining changes in intrahemispheric networks are revealing insights into possible compensatory reorganization in early PD to maintain motor function, which eventually fails as dopaminergic systems degenerate [39,40]. PD patients demonstrate reductions in interhemispheric functional connectivity across sensorimotor cortices (precentral, postcentral, paracentral lobule), posterior parietal regions (superior parietal lobule, supramarginal gyrus, precuneus), and temporal–occipital areas, as measured by voxel-mirrored homotopic connectivity (VMHC). The interhemispheric functional connectivity decreases in parallel with structural connectivity reductions in the same regions and is accompanied by decreased fractional anisotropy in the corpus callosum connecting these areas, suggesting that compensatory mechanisms must operate within a framework of compromised structural interhemispheric communication [39]. The ability to dynamically transition between network states appears critical for compensation. Early PD patients show lower state attendance for globally integrated network states and fewer total transitions between states, with worse motor scores and greater dopaminergic impairment associated with reduced average dwell time in globally integrated states and fewer transitions. Preservation of motor performance in early PD cohorts may be linked to maintaining dynamic engagement and disengagement of interconnected brain states, specifically through bidirectional transitions between globally integrated and lesser-connected states [40]. These types of studies using fMRI are helping to form insight into changes that occur during neurodegenerative diseases such as PD, which will lead to not only improved understanding of the disease process but will lead to improvement of biomarkers to follow effects of neuroprotective therapies.

Nigrostriatal pathway degeneration in Parkinson’s disease interacts with limbic circuits through combined dopaminergic and serotonergic denervation that differentially affects distinct neural networks, with apathy primarily resulting from mesolimbic dopaminergic dysfunction affecting reward processing, while depression involves broader serotonergic alterations in limbic–cortical circuits, particularly the anterior cingulate cortex. In apathy, severe dopamine depletion in the mesocorticolimbic system impairs emotional reactivity and reward-related learning, particularly affecting circuits connecting the ventromedial prefrontal cortex with the amygdala and nucleus accumbens. This dopaminergic denervation disrupts the ventral striatum–medial prefrontal cortex connection, leading to impaired incentive processing and blunted emotional responses [41]. Depression in Parkinson’s disease involves a more complex interaction. While dopaminergic dysfunction contributes, serotonergic degeneration appears to play a more prominent role. Functional connectivity studies suggest that depression in Parkinson’s disease is associated with increased ventral tegmental area–anterior cingulate cortex connectivity, while apathy shows decreased caudate–thalamus and orbitofrontal–parahippocampal connectivity [35]. Recent neuroimaging and biomarker studies suggest distinct neural signatures that differentiate apathy from depression in Parkinson’s disease, where apathy appears primarily linked to striatal dopamine transporter (DAT) decline and noradrenergic locus coeruleus degeneration, while depression involves limbic serotonergic dysfunction [41,42].

## 5. Management Strategies

Currently, there are no cures for PD or neuroprotective treatments that may lessen cell loss, thereby slowing or halting disease progression. Hundreds of substances have been studied in the search for neuroprotective treatments. Unfortunately, all have failed, likely in part due to the fact that up to 75% of the dopaminergic neurons in the substantia nigra have lost function by the time of diagnosis in the early stages of the disease. Therefore, starting a potential neuroprotective agent at the time of diagnosis of motor symptoms may be too late in the degenerative intracellular cascade of processes contributing to neuronal cell loss [23,29,30]. Thus, the need for identification of earlier biomarkers is undergoing extensive research. When providing treatment to individuals with mood disorders, PD, and both, it is well recognized that they will exhibit widely variable symptoms with variable severities. Therefore, it is imperative to tailor treatment to the individual. A variety of pharmacologic and non-pharmacologic therapies are available.

### 5.1. Non-Pharmacological Interventions (Table 1)

Non-pharmacological interventions will be beneficial for mood symptoms as well as PD-specific symptoms. Exercise and physical therapy are universally recommended as essential components of PD management, with benefits extending to both motor and non-motor symptoms. Structured exercise programs, including aerobic, resistance, and stretching exercises, have demonstrated improvements in mobility, gait, and balance, as well as reductions in psychological distress [43,44]. Mind–body interventions such as yoga and tai chi are increasingly recognized for their dual physical and psychological benefits. In a randomized clinical trial, mindfulness yoga was shown to be as effective as stretching and resistance training in reducing anxiety and depression in PD patients [44]. These interventions are not only enjoyable and culturally adaptable but also economically feasible, making them particularly valuable in resource-limited settings [45]. Cognitive behavioral therapy (CBT) is the most extensively studied and recommended non-pharmacological intervention for PD patients with comorbid depression and anxiety. Meta-analyses demonstrate that CBT, whether delivered individually, in groups, or via telemedicine, leads to significant reductions in both depressive and anxiety symptoms. Alnajjar et al. reported standardized mean differences (SMDs) of −1.02 for depression and −0.95 for anxiety, with both traditional and tele-CBT modalities showing substantial efficacy [46]. Wu et al. found that CBT not only improves mood symptoms but also enhances cognitive function and overall quality of life in PD patients (mean difference in QoL: 3.45, 95% CI 1.13–5.57, *p* = 0.04) [47]. Group CBT has also been validated, with evidence showing reductions in anxiety and depressive symptoms and improved adherence to treatment regimens [48]. CBT protocols for PD are often adapted to address disease-specific concerns, such as coping with motor fluctuations, loss of independence, and existential distress. The involvement of caregivers in CBT programs is common and may further enhance outcomes [49]. The evidence and clinical practice experience both support the use of CBT as a routine part of multidisciplinary care for PD patients with psychiatric comorbidities [50]. While CBT is most robustly supported for depression and anxiety, preliminary data suggest potential benefits for impulse-control disorders and insomnia [49].

**Table 1:** Non-pharmacologic interventions for Parkinson disease with comorbid mood disorders.

Intervention	Primary Targets	Evidence-Based Benefits	Clinical Implementation Notes
**Cognitive** **Behavioral** **Therapy** **(CBT)**	Depression, anxiety, coping with chronic illness	Reduces depressive and anxiety symptoms; improves coping, adherence, and quality of life	Can be delivered in in-person, group, or telehealth format; tailor to PD-specific stressors (motor fluctuation, loss of independence)
**Exercise and Physical** **Therapy**	Motor function, mood, fatigue, cognition	Improves mobility, balance, and non-motor symptoms; reduces depression and apathy	Aerobic and resistance training recommended ≥3× weekly; supervised programs enhance adherence
**Mindfulness, Yoga, and** **Tai Chi**	Anxiety, stress, motor control, sleep	Enhances mood regulation, reduces stress, improves postural stability and sleep quality	Effective as adjunctive therapy; mindfulness yoga comparable to CBT in mood improvement
**Occupational and Speech Therapy**	Functional independence, communication, cognition	Improves daily functioning, speech clarity, and social interaction; supports cognitive engagement	Early referral promotes adaptation and prevents isolation
**Caregiver** **Education and Support**	Caregiver burden, patient–family dynamics	Improves patient outcomes and reduces caregiver distress	Incorporate caregiver participation into treatment planning and CBT sessions
**Multidisciplinary Care** **Coordination**	Integrated symptom management, safety, QoL	Enhances outcomes across motor, cognitive, and mood domains; reduces hospitalizations	Optimal model includes neurologist, psychiatrist, nurse, PT/OT, and social worker

Abbreviations: CBT = cognitive behavioral therapy; PT/OT = physical and occupational therapy; QoL = quality of life.

### 5.2. Pharmacological Treatment Strategies (Table 2)

The treatment of PD symptoms and mood is not necessarily different per se than treating patients without these coexisting morbidities in that the same medications are used. However, due to the mood and PD comorbidity, there are potential increased risks of side effects [23,43,50,51]. Also, symptom control of one condition may affect control of the other condition. This will complicate decisions regarding which condition and medications to adjust. For example, if a patient has depression and PD, if the depression is exacerbated or “comes out of control,” the PD tremor or bradykinesia may worsen. If the worsening depression is not identified and the focus is on treatment of the PD symptoms resulting in higher doses of PD medications, the patient may not have or perceive improvement in their motor PD symptoms. A negative thought process may arise in which the patient thinks that “their depression will improve if their PD improves.” However, if the depression is recognized and treated, the “PD symptoms” may improve as well. Insightful evaluation of symptoms is essential.

**Table 2:** Pharmacologic management of Parkinson disease and comorbid mood disorders.

Medication Class	Examples	Primary Indication	Key Clinical Considerations in PD + Mood Disorders	Notable Adverse Effects/Cautions
**Dopaminergic therapy**	Levodopa/carbidopa, benserazide/levodopa	Motor symptom control	First line for bradykinesia, rigidity, tremor; may modestly improve mood; adjust dosing gradually	Dyskinesia, nausea, orthostatic hypotension, hallucinations with long-term use
**Dopamine agonists**	Pramipexole, ropinirole, rotigotine	Motor symptom adjunct or monotherapy in early disease	Useful for reducing “off” time; may have mild antidepressant effect	Impulse-control disorders, hallucinations, somnolence, mania in bipolar patients
**MAO-B inhibitors**	Selegiline, rasagiline, safinamide	Motor symptom adjunct; mild antidepressant effect	Can be combined with levodopa; avoid combining with SSRIs/SNRIs due to serotonin syndrome risk	Insomnia, hypertension, serotonin syndrome (with serotonergic drugs)
**COMT inhibitors**	Entacapone, opicapone, tolcapone	Prolong levodopa effect, reduce “off” periods	Improves motor fluctuations; adjust to minimize polypharmacy	Diarrhea, hepatotoxicity (tolcapone), dyskinesia
**Antidepressants (SSRIs)**	Sertraline, citalopram, escitalopram, paroxetine	Depression, anxiety	First line for mood symptoms; generally motor-neutral	Worsened tremor in some cases, GI upset, hyponatremia, serotonin syndrome with MAO-B inhibitors
**Antidepressants (SNRIs)**	Venlafaxine, duloxetine	Depression, anxiety, pain	May improve energy and concentration; modest dopaminergic benefit	Tremor exacerbation, hypertension, withdrawal symptoms
**Tricyclic antidepressants (TCAs)**	Nortriptyline, desipramine	Depression (refractory cases)	Effective but use cautiously in older PD patients	Anticholinergic effects, arrhythmia, orthostatic hypotension
**Mood stabilizers**	Lithium, valproate, lamotrigine	Bipolar disorder, mood stabilization	Lamotrigine preferred for minimal motor worsening; lithium/valproate may exacerbate tremor or rigidity	Tremor, ataxia, sedation, drug–drug interactions
**Atypical antipsychotics**	Quetiapine, clozapine, pimavanserin	Psychosis, bipolar disorder	Quetiapine and clozapine are preferred due to minimal motor worsening; pimavanserin approved for PD psychosis	Sedation, orthostatic hypotension, agranulocytosis (clozapine), QT prolongation
**Anxiolytics**	Buspirone, clonazepam (RBD only)	Anxiety, REM sleep behavior disorder	Limited evidence for anxiety; clonazepam effective for RBD; avoid chronic benzodiazepine use	Sedation, falls, cognitive impairment, tolerance

Abbreviations: MAO-B = monoamine oxidase type B; COMT = catechol-O-methyltransferase; SSRI = selective serotonin reuptake inhibitor; SNRI = serotonin–norepinephrine reuptake inhibitor; RBD = REM sleep behavior disorder.

#### 5.2.1. Treatment of Motor Symptoms

The pharmacological management of PD in patients with pre-existing depression, anxiety, or BPAD requires careful selection of agents, attention to drug interactions, and individualized dosing. The mainstay for treating the motor symptoms of PD is dopamine replacement. Several medications are available that enhance or “replace” brain dopamine levels. The oldest and best dopamine replacement options are the levodopa formulations [10,11,12]. Levodopa needs to be given with a decarboxylase inhibitor, either carbidopa or benserazide, to minimize peripheral metabolism, which helps reduce peripheral side effects and improves bioavailability and absorption across the blood–brain barrier [12]. Levodopa will reduce rigidity and bradykinesia and often dampens tremors. It usually will not help to reduce non-motor symptoms. Initially, patients may have a dramatic response and feel “back to normal.” The duration of this “on” time with good symptom improvement will be several hours. As the disease progresses, the “on” time will shorten and the patient will experience times in which their symptoms intrude (“off” time). This type of motor fluctuation is likely due to loss of dopaminergic neurons causing decline of the endogenous dopamine levels, short half-life of levodopa, and inconsistent intestinal absorption [10,11,23,51,52,53].

Other medications that can reduce motor symptoms include dopamine agonists (either as monotherapy or in addition to levodopa). Dopamine agonists (pramipexole, ropinirole, rotigitine) activate receptors in the striatum and have a longer half-life, which may provide better basal levels and activity, thereby lessening fluctuations. However, dopamine agonists are more difficult to tolerate and do not work as robustly in reducing motor symptoms [23,51,52,53]. Catechol-O-methyltransferase (COMT) inhibitors or monoamine oxidase B (MAO-B) inhibitors can enhance dopamine activity by interfering with the metabolic pathways of dopamine. Use of agents affecting other neurotransmitter systems (NMDA receptor blockers, adenosine receptor antagonists, anticholinergics) can provide benefit as well [23,51,52,53].

All of the dopaminergic agents can produce significant side effects, both short-term and long-term, so the judicious use of these drugs is imperative. Thoughtful polypharmacy may be necessary. Most clinicians practice the philosophy of “start low and go slow”; starting at low doses, increasing slowly, and stopping at the lowest effective dose. Patients may experience nausea, sedation, orthostatic hypotension, and visual hallucinations from dopaminergic medications. With long-term use, many will develop levodopa-induced dyskinesias, which can include choreiform movements and dystonic spasms. Often, adjusting the dose resolves these dyskinesias. Since PD is a progressive disease, dosing and medication schedules will need to be adjusted and personalized to the individual. In some situations, the patient can be taught to self-adjust medications based on symptoms; in other situations, a rigid dosing schedule may be necessary. Balancing symptom control while minimizing side effects is an ongoing process. With advanced disease, some may develop treatment-resistant symptoms, especially balance and gait issues. As PD progresses, medications will need to be adjusted to keep the patient in an “on” state with the fewest side effects possible. This is where the various formulations of levodopa (regular-release, longer-acting formulations, inhaled forms, subcutaneous infusions, intra-intestinal infusions) may be useful [23,51,52,53].

In some cases, such as medically refractory tremors and severe motor fluctuations, surgical treatments may be reasonable. Currently, deep brain stimulation (DBS) has become a standard of care at most major movement disorders centers [23,52]. Also, ablative surgeries (MRI-guided focused ultrasound or stereotactic radiosurgery) are becoming increasingly available [54,55]. Of course, these procedures have their own associated risks, and patient selection is key to a successful outcome. These procedures are not curative, or neuroprotective; however, they can reduce symptoms, thereby improving quality of life for significant spans of time in appropriately selected patients.

#### 5.2.2. Treatment of Mood

Early after diagnosis of PD, people often experience a grief response, which could precipitate worsening of their pre-existing mood disorder. Some people may experience worsening of their mood from the stress of living and coping with a new life-altering diagnosis. Furthermore, the neurochemical changes that occur as PD progresses may affect mood. Consequently, close monitoring of mood issues is important at the time of diagnosis and as the disease progresses.

For depression and anxiety, selective serotonin reuptake inhibitors (SSRIs) such as citalopram, escitalopram, paroxetine, fluoxetine, and sertraline, as well as serotonin–norepinephrine reuptake inhibitors (SNRIs) like venlafaxine, are considered first-line agents [32,56,57]. Tricyclic antidepressants (TCAs), particularly nortriptyline and desipramine, have demonstrated efficacy in PD-related depression, with some meta-analyses suggesting a more favorable balance between efficacy and acceptability compared to SSRIs (SMD for TCAs vs. placebo: −0.83, 95% CI −1.53 to −0.13) [58] However, TCAs carry a higher risk of anticholinergic side effects, orthostatic hypotension, and cardiac conduction abnormalities, which may be particularly problematic in older PD patients or those with autonomic dysfunction [56,57,58,59]. The choice between SSRIs, SNRIs, and TCAs should be individualized, considering comorbidities, side-effect profiles, and potential interactions. Bupropion (norepinephrine–dopamine reuptake inhibitor) formulations are often deemed optimal for treatment due to dopamine-enhancing properties; however, they often enhance tremors.

Dopamine replacement can benefit mood variably in patients with PD; however, it is usually not the main treatment for mood due to variable and inconsistent efficacy. Dopamine agonists, notably pramipexole, have shown antidepressant effects in PD and may be considered in patients with mild-to-moderate depressive symptoms, especially when motor symptoms also require optimization (OR for efficacy vs. placebo: 2.20, 95% CI 1.46–3.33) [56,57]. However, dopamine agonists can exacerbate impulse-control disorders, psychosis, and, in rare cases, mania, necessitating caution in patients with bipolar affective disorder or a history of psychosis [60,61]. Selective MAO-B inhibitors such as selegiline and rasagiline have demonstrated efficacy for depressive symptoms in PD [57]. These agents may be considered, particularly when motor symptoms also require adjunctive therapy. Some sources caution that combining MAO-B inhibitors with SSRIs, SNRIs, or TCAs can increase the risk of serotonin syndrome [56,57,58,59]; however, other studies have shown little if any risk when used at the recommended low therapeutic doses [62,63]. Clinical experience has shown that prescribing rasagiline or selegeline with SSRIs, SNRIs, TCAs, or bupropion is quite well tolerated and the risk serotonin syndrome is exceedingly low. Of course, good clinical practice supports continual monitoring of individuals for any side effects when initiating and adjusting medication regimens.

Treatment of anxiety is complex, with few trials of specific agents. Many people with PD will experience wearing-off anxiety, which can be treated by optimizing the dopaminergic therapy. Other options for treatment of anxiety are SSRIs and SNRIs. Benzodiazepines, such as clonazepam, may be used for specific indications like REM sleep behavior disorder, but their use for anxiety is limited by risks of sedation, cognitive impairment, and falls, especially in older PD patients [37,52,64]. Unfortunately, there are few randomized controlled trials of treatment of anxiety in PD. Buspirone (a serotonin 1A [5-HT_1A_] partial agonist) is often used for treatment of anxiety in the general population; however, use of this medication is often not well tolerated in the PD population [65]. Non-pharmacologic therapies may be more effective strategies.

Management of PD in patients with pre-existing BPAD is particularly challenging due to the risk of mood destabilization by dopaminergic agents and antidepressants. Dopamine agonists and levodopa can precipitate or exacerbate manic symptoms, and antidepressants may induce mania or rapid cycling. In such cases, mood stabilizers (e.g., lithium, valproate, lamotrigine) may be required, but these agents have limited evidence in PD and may interact with antiparkinsonian drugs [16,28]. Furthermore, both lithium and valproate have been implicated in medication-induced parkinsonism. There are many case reports describing valproate-induced parkinsonism. A critical review identified 116 published cases, with prevalence rates ranging from 1.4% to 75% among valproate users, though most cases occurred at therapeutic plasma concentrations. Parkinsonism was typically reversible after discontinuation, but recovery could be prolonged or incomplete. Dopaminergic deficits were confirmed in some cases, and a subset of patients responded to dopaminergic therapy [66,67,68]. Although lithium is less commonly implicated in drug induce parkinsonism, there is epidemiological and case-based support for a possible association. Case series and cohort studies have reported lithium as a cause of parkinsonism, sometimes at therapeutic levels. In one study, lithium was the suspected cause in 15% of drug-induced parkinsonism cases, with normal DaT scan imaging in most, suggesting a non-degenerative mechanism. A large retrospective cohort study found that lithium monotherapy for at least one year was associated with an increased incidence of subsequent dopaminergic drug prescribing (adjusted hazard ratio 1.68–1.87) compared to antidepressant users, though diagnostic codes may not distinguish idiopathic PD from other parkinsonian syndromes [28,69]. In the author’s experience, if the individual’s BPAD has been stable for at least 6 months, it is reasonable to start a trial of a dopaminergic medication if motor symptoms are significantly impairing daily function (i.e., tremor or rigidity interfering with daily activities such as brushing teeth, keyboarding, rising from a chair, or walking). The longer the individual’s mood has been stable, the lower the chance of mood destabilization. Carbidopa/levodopa or its equivalent is the preferred medication as it will usually have the most robust effect on reducing motor symptoms with the fewest side effects [51]. If mania or depression occurs, immediate dose reduction or discontinuation is recommended. For individuals with suspected valproate- or lithium-induced PD, it is preferable to slowly reduce those agents if possible. When tapering valproate or lithium, it may take 6–12 months before PD symptoms improve [60,69]. Every medication change needs to be individualized, performed in a systematic fashion, and monitored carefully. Making a lot of simultaneous changes is not recommended as responses and side effects cannot be appropriately tracked.

For treatment of psychotic symptoms such as visual hallucinations or delusions, quetiapine and clozapine are preferred due to their lower propensity to worsen parkinsonism, with clozapine requiring regular hematological monitoring. Pimavanserin, a selective serotonin 5-HT_2A_ inverse agonist, is FDA-approved for PD psychosis and does not worsen motor symptoms, but may only be reduce mild visual hallucinations [16,28,43,60,61]. Naturally, drug interactions are a major concern. Dopamine agonists and levodopa can exacerbate psychosis and impulse-control disorders and may precipitate mania in bipolar patients. Antipsychotics with strong D2 antagonism (e.g., risperidone, olanzapine, haloperidol) should be avoided due to the risk of worsening motor symptoms [28,43,60,61].

## 6. Monitoring, Adverse Effects, and Multidisciplinary Care

The long-term management of PD in patients with pre-existing depression, anxiety, or BPAD requires a highly structured approach to monitoring and mitigating adverse effects and drug interactions. The risk of adverse effects and drug interactions is heightened in PD patients with psychiatric comorbidities due to necessary polypharmacy. The selection of pharmacological agents should be individualized, prioritizing medications with the most favorable efficacy-to-tolerability ratio and the lowest interaction potential. Regular monitoring for adverse effects is critical, especially during medication initiation and titration and after any changes in the regimen. Key adverse effects to monitor include anticholinergic burden, orthostatic hypotension, impulse-control disorders, QT prolongation, cardiac arrhythmias, and agranulocytosis with clozapine [57,60,61].

Whenever possible, the medication regimen should be simplified to minimize polypharmacy and reduce the risk of adverse effects and interactions. This may involve using the lowest effective doses, avoiding unnecessary adjunctive agents, and discontinuing medications that are not providing clear benefit or are contributing to adverse effects. In cases of psychosis or severe neuropsychiatric symptoms, the first step is often to reduce or discontinue medications that may be contributing (e.g., anticholinergics, amantadine, dopamine agonists), while balancing the risk of worsening motor symptoms [23,28,43,60,61,64].

Optimal management requires a multidisciplinary approach, involving neurology, psychiatry, pharmacy, primary care, physical and occupational therapy, speech pathology, neuropsychology, and social work. Regular follow-up visits should include assessment of both motor and non-motor symptoms, medication side effects, and functional status. Patient and caregiver education are essential to ensure early recognition of adverse effects and to promote adherence [37,61,64]. Non-pharmacological interventions, such as cognitive behavioral therapy and exercise, should be integrated to reduce reliance on medications and mitigate neuropsychiatric symptoms [43,44,45].

Multidisciplinary care models and coordinated care pathways have a demonstrable positive impact on clinical outcomes and quality of life for PD patients with psychiatric comorbidities. Randomized controlled trials and systematic reviews provide direct evidence that multidisciplinary care models improve both clinical outcomes and quality of life in PD patients, including those with psychiatric comorbidities [70,71,72,73,74]. In a randomized trial, van der Marck et al. demonstrated that patients managed by a specialist multidisciplinary team experienced significant improvement in quality of life (mean improvement in PDQ-39 of 3.4 points, 95% CI 0.5–6.2), motor function, depression, psychosocial functioning, and reduced caregiver strain compared to those receiving standard neurologist care [70]. The presence of a dedicated care coordinator—such as a PD nurse or case manager—has emerged as a critical facilitator of effective multidisciplinary care [74].

The care team members may vary depending on the patient’s needs. Often, physical therapy is extremely beneficial. The physical therapist is able to tailor an exercise program that not only focuses on reducing the patient’s PD symptoms, but also considers any limitations imposed by musculoskeletal conditions. For example, someone with PD and significant hip arthritis may need to modify particular exercises. Additionally, physical therapists will be knowledgeable of local exercise classes and can advise an individual on which ones will be safe and beneficial for them. Occupational therapists can assist with improving the ease of daily activities, especially if the patient is starting to become physically limited. Occupational therapists and speech pathologists can help to gain insight into cognitive function and how that may be affecting the patient’s daily activities. To help assessment of the interaction between mood dysfunction and cognitive loss, neuropsychological testing may provide benefit. Occupational therapists, speech pathologists, and neuropsychologists can help to provide strategies for coping with cognitive issues. People with PD often have difficulties swallowing, and the speech pathologist will be invaluable in assessing swallow function and providing strategies to compensate, thereby lessening the risk of aspiration events and pneumonia. Also, people with PD often have voice issues (hypophonia), which makes communication difficult, which can often contribute to social isolation and exacerbation of baseline mood difficulties. Speech pathologists will be able to assist with voice exercises and offer strategies to maintain and optimize communication skills. Social workers can help the patient and care team find the best community resources and support systems (i.e., support networks, in-home assistance programs, disability resources, etc.) [23,70,71,72,73].

The most effective strategies for monitoring and managing adverse effects and drug interactions in PD patients with pre-existing depression, anxiety, or BPAD involve comprehensive baseline and ongoing assessment, rational drug selection and regimen simplification, vigilant monitoring for adverse effects and drug interactions, multidisciplinary care, and patient/caregiver education. These strategies are supported by current evidence and expert consensus.

## 7. Conclusions

PD occurring in individuals with pre-existing mood disorders presents a complex diagnostic and therapeutic challenge with important clinical and public health implications. Symptom overlap, medication interactions, and bidirectional neuropsychiatric effects can delay recognition and complicate management, underscoring the need for systematic screening, careful longitudinal assessment, and integrated care models. Evidence and clinical experience support the use of structured diagnostic approaches and individualized treatment strategies that simultaneously address motor and non-motor symptoms while minimizing psychiatric destabilization.

From a public health perspective, earlier identification of PD in high-risk psychiatric populations improves cross-disciplinary collaboration, and a greater awareness of neuropsychiatric comorbidity can reduce morbidity, functional decline, and healthcare burden. Practitioners and policy makers should recognize the urgent need for multidisciplinary care models and workforce development that integrate neurology and mental health services to address complex comorbidities more effectively. Delivery-of-care systems need to be developed to improve access to underserved areas including telemedicine infrastructure, consulting networks, and pharmaceutical programs to ensure access to essential medications (levodopa, antidepressants), thereby removing financial barriers that disproportionately affect vulnerable populations.

Future research should prioritize funding for studies on modifiable risk factors, early biomarkers, and basic neuroscience. Basic neuroscience research is key to improving our understanding of the pathologic mechanisms of neurodegenerative disease. The information gained from this research will lead to the continued development of diagnostics and treatments. All of these efforts will ultimately lead to reductions in healthcare burden globally as well as for the individual.

## Data Availability

No new data were created or analyzed in this study.
